# Emerging Antimicrobial and Immunomodulatory Fiber-Based Scaffolding Systems for Treating Diabetic Foot Ulcers

**DOI:** 10.3390/pharmaceutics15010258

**Published:** 2023-01-11

**Authors:** Helena P. Felgueiras

**Affiliations:** Centre for Textile Science and Technology (2C2T), University of Minho, Campus de Azurém, 4800-058 Guimarães, Portugal; helena.felgueiras@2c2t.uminho.pt; Tel.: +351-253-510-289

**Keywords:** diabetes, chronic wounds, fiber-based scaffolds, bioactive agents, antimicrobial and immunomodulatory functions, improved wound healing

## Abstract

Diabetic foot ulcers (DFUs) are one of the main complications of diabetes and are characterized by their complexity and severity, which are frequently aggravated by overexpressed inflammatory factors and polymicrobial infections. Most dressing systems offer a passive action in the treatment of DFUs, being frequently combined with antibiotic or immunomodulatory therapies. However, in many instances due to these combined therapies’ inability to properly fight microbial presence, and provide a suitable, breathable and moist environment that is also capable of protecting the site from secondary microbial invasions or further harm, aggravation of the wound state is unavoidable and lower limb amputations are necessary. Considering these limitations and knowing of the urgent demand for new and more effective therapeutic systems for DFU care that will guarantee the quality of life for patients, research in this field has boomed in the last few years. In this review, the emerging innovations in DFU dressing systems via fiber-based scaffolds modified with bioactive compounds have been compiled; data focused on the innovations introduced in the last five years (2017–2022). A generalized overview of the classifications and constraints associated with DFUs healing and the bioactive agents, both antimicrobial and immunomodulatory, that can contribute actively to surpass such issues, has also been provided.

## 1. Introduction

Diabetes Mellitus (DM) was one of the first illnesses to be identified, in which the recognition of symptoms by humankind has been generally understood. Because of its high prevalence, and high morbidity and mortality rates (among the top ten causes of death worldwide), DM has been deemed a global epidemic [1]. Among USA adults prevalence has been increasing overtime, above global rates, from 5.3% in 1976–1980 to 11.5% in 2011–2014, with economic costs reaching 24.0% of the total health care annual budget [2]. Diabetic foot ulcers (DFUs) are one of the main complications of DM, with important health, physical, mental, social, and economic impacts. The average global prevalence of DFUs is ≈6.3%, with North America rating at the top with 13.0% prevalence, and Oceania at the bottom with 3.0% [3]. In DM patients, the development of DFUs is a lifetime risk estimated at 25%, with wound recurrence being a very likely possibility [4]. DFUs are categorized as chronic wounds in which the loss of the epidermis and dermis is frequently followed by subcutaneous and underlying tissue exposure. Additionally, because of their location, they are highly susceptible to infections, which can spread very quickly, compromising the neighboring healthy tissues and in advanced cases or scenarios of ineffective/deficient care, leading to systemic infections and even limb loss (amputation). In fact, it is estimated that >70% of DFU patients may require lower limb amputation due to health complications, poor wound management, and treatment ineffectiveness [5]. The multifactorial pathophysiology that characterizes DFUs, namely diabetic neuropathy (DN), peripheral arterial disease and immunosuppression with an exacerbated inflammatory response, makes management and treatment selection very complex [6].

As in most diseases, early diagnoses of DFUs and treatment selection are key. Standard therapies include glycemic control, debridement, infection management and ulcer off-loading. However, as the wound progresses to more complex and difficult-to-treat stages, advanced treatment options are required [7]. It is in this category that many antimicrobial and immunomodulatory formulations based on fibrous constructs are making their mark. This review explores this further, by exposing the emerging innovations in DFU care via fiber-based scaffolding systems modified with bioactive compounds. The work provides a generalized overview of the classifications and constraints associated with DFU healing, identifies effective bioactive agents used to fight microbial infections or regulate immunomodulatory pathways and introduces emerging fiber-based formulations that are promising for DFU care (focusing on the last five years). 

## 2. Diabetic Ulcers: Healing Impairments and Classifications

### 2.1. Impairments

**Figure 1:**
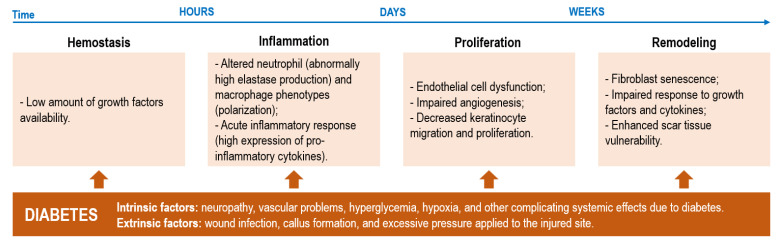
Possible disruptive events occurring within each phase of healing and the intrinsic and extrinsic diabetic factors that contribute to such disturbances in the normal healing cascade of wounds (adapted with CC BY 4.0 permission from [9]).

DFUs are complex wounds that affect the lower extremities of diabetic patients. Even though DFUs are initially characterized as acute wounds, their inability to progress through the healing stages, frequently taking 12 or more weeks to properly heal (generally, acute wound healing is completed within 4 to 6 weeks), converts them into chronic wounds [4,5,8]. Ulcerations at the lower extremities are frequently associated with neuropathic pain and peripheral arterial disease with the healing cascade being largely affected by overexpressed inflammatory factors (prolonged inflammation), hyperglycemia, hypoxia, deregulated enzyme activity and alterations in various signaling pathways, including the activity of neuropeptides. Additionally, healing progression is also hindered by external influences, particularly the presence of pathogens (Figure 1) [9].

**Figure 2:**
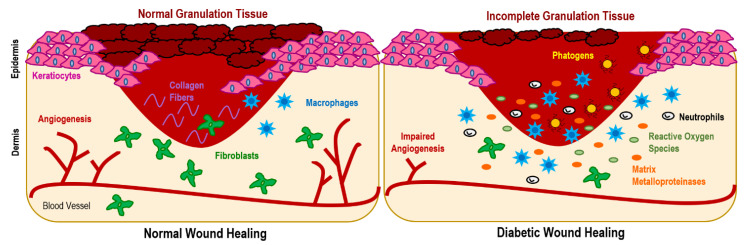
Diabetic wounds are characterized by impaired angiogenesis, excessive inflammation (increase in macrophage recruitment), augmented production of matrix metalloproteinases, hypoxia and hyperglycemia, which is associated with a raise of the reactive oxygen species expression that prevents the formation of healthy tissue. All these factors condition granulation tissue formation, maintaining the wound in an inflammatory state, which is also exacerbated by installation of pathogenic microbes.

It is general knowledge that diabetes is associated with high glucose levels in the blood. In DFUs, these abnormally high amounts of glucose aside from lowering growth factor availability and hindering leucocyte recruitment for fighting infection can also lead to vasoconstriction and microvascular dysfunctions that limit tissue oxygenation. By restricting oxygen supply (also instigated by neuropathy damage in local nerves), hypoxia scenarios may be triggered. Hypoxia increases the number of free radicals which, in combination with enhanced pro-inflammatory cytokine expression, further delays healing [10,11]. Additionally, a dysregulated M1 and M2 macrophage ratio due to the inability of macrophages to polarize from the pro-inflammatory M1 phenotype to the anti-inflammatory M2 phenotype following inflammation has been shown to stall DFUs healing at this phase. At this stage, the expression of anti-inflammatory markers, such as interleukin-12 (IL-12), IL-6, IL-1 or tumor necrosis factor-α, is enhanced, while the activity of anti-inflammatory cues, such as IL-10 and IL-4, is reduced [7,12]. This leads to an imbalance between angiogenic factors (promoters of new blood vessel formation) and angiostatic factors, impairing angiogenesis and leading to endothelial progenitor cell dysfunction aside from hindering the proliferation and migration of fibroblasts and keratinocytes [10,13]. This is exacerbated by the limited neuropeptide activity at the site, since in hyperglycemia conditions, neuropeptides such as Substance P, neuropeptide Y or neurotensin endowed with regenerative functions, have their activity reduced. Additionally, matrix metalloproteinases (MMPs), which are responsible for promoting autolytic debridement and cell migration in acute wound scenarios, entering in remission after inflammation, become overly expressed in DFUs degrading various matrix proteins, growth factors and blocking cell proliferation [7,14]. All these events (Figure 2), exacerbate wound chronicity, conditioning granulation tissue formation.

Aside from the previous phenomena, the increased incidence of infections in DFUs contributes significantly to impaired healing. In fact, DFUs are more prone to develop environments conducive to microbial installation than other types of chronic wounds due to the inherent stress and compressive forces that characterize the foot area, which has been known to promote the overgrowth of bacteria [14]. Additionally, due to DFUs-associated neuropathy, infection progression and inherent symptoms are more difficult to detect, delaying a proper treatment response that may allow microbes to settle and colonize the injured site. Further, even in a normal scenario, the foot microflora are very proliferative and varied. Thus, skin commensal bacteria can also colonize the wound generating a self-produced protective extracellular biofilm that may become pathogenic and immune to all antimicrobial events triggered by the healing cascade [15]. Among the many microorganisms colonizing DFUs, the most prevalent are *Staphylococcus aureus*, β-hemolytic streptococci, *Enterobacteriaceae* and various anaerobes [16].

### 2.2. Classification

Various classification systems have been proposed to identify the severity of DFUs. The most employed and reliable is the Wagner Ulcer Classification System established in 1981, which assesses ulcer depth and the presence of osteomyelitis or gangrene [17]:

- Grade 1. Partial thickness involving only dermis and epidermis
- Grade 2. Full thickness and subcutaneous tissues
- Grade 3. Grade 2 plus exposed tendons, ligament, and/or joint
- Grade 4. Grade 3 plus abscess and/or osteomyelitis
- Grade 5. Grade 3 plus necrotic tissue in wound
- Grade 6. Grade 3 plus gangrene in the wound and surrounding tissue

Another option is by following the classification introduced by the University of Texas, which categorizes DFUS based on the occurrence of ischemia or infection and ulcer depth. Additional classification systems have been proposed over the years, although the first remains the most generally accepted [7].

## 3. Antimicrobial Agents

Most DFUs are infected with pathogenic microorganisms. Infected DFUs result from the entrance, growth, metabolic activities, and propagation of microorganisms at the injured site and surrounding tissues, which trigger pathophysiological effects. Here, microorganisms can either colonize the wound, multiply on the surface of the wound, or invade the underlying tissues, actively penetrating the soft tissues around the ulcer. Yet, the presence of microorganisms in the DFUs is not as important as the rate of pathogen growth. Many reports have shown that an infected wound is only labeled as such if the number of microorganisms per g of tissue surpasses 100,000 [18]. To prevent that from occurring and/or to combat infection once this boundary is surpassed, many antimicrobial agents have been examined and included within antimicrobial-fighting strategies for DFUs care: antibiotics, natural extracts, antimicrobial peptides, organic and inorganic nanoparticles, bioactive polymers, etc.

### 3.1. Antibiotics

Antibiotics can be chemically synthesized or extracted from microbial substances. Their effectiveness is defined based on their ability to target elements within the bacterial cell wall and intracellular space and induce a detectable effect without losing their effectiveness throughout the process [19]. Studies have reported the ability of bacteriostatic (prevent bacterial growth) and bactericidal (kill bacteria) antibiotics to assist with wound closing by effectively acting against infection-producing microbes. They accomplish such deeds by targeting the bacteria cell wall biosynthesis, hindering protein synthesis or inhibiting nucleic acid replication [20]. Indeed, in DFU infection treatment strategies, doxycycline, a broad-spectrum tetracycline antibiotic and matrix metalloproteinases inhibitor, has been shown to hinder *Escherichia coli* bacterium activity by means of a polylactide nanofiber-based delivery system [21]. Vancomycin (glycopeptide antibiotic) and gentamicin (aminoglycoside antibiotic) have also contributed significantly to control infection and instigate regeneration when loaded onto co-axial sheath-core nanofibrous poly(lactide-co-glycolide) scaffolds modified with platelet-derived growth factors [22]. Additionally, core-shell nanofibers composed of polyethylene oxide, chitosan and vancomycin at the shell and polyvinylpyrrolidone, gelatin and imipenem/cilastatin (β-lactams) at the core have demonstrated these antibiotics effectiveness in eliminating *Escherichia coli*, *Pseudomonas aeruginosa* and multi-resistant *Staphylococcus aureus* bacteria colonizing DFUs [23]. Most importantly synergisms between antibiotics have been revealed, so that the amount loaded within the fibrous scaffolds required for effective action could be reduced and potential side effects against viable tissues mitigated [24]. Even though antibiotics continue to be used in DFU care, their repeated and/or improper usage has been known to increase bacterial resistance [19]. In fact, ≈ 70% of bacteria responsible for wound infections are resistant to at least one type of antibiotic, with many infectious strains revealing resistance against many [25].

### 3.2. Natural Extracts

Natural extracts are obtained from plants, particularly from their secondary metabolites, which have been identified as most effective in fighting pathogen-derived infections. Phenolic compounds are produced by plants in response to bacterial and fungal attacks and possess a phenol moiety in their structure. They are made of one (phenolic acids) or more (polyphenols) aromatic rings with attached hydroxyl groups. They act against microbial cells by generating non-specific interactions with proteins or by inhibiting the action of their enzymes [19,26]. Terpenes include one or more five-carbon isoprene units in their structure (the largest class of secondary metabolites found in essential oils), acting against pathogens by lipophilic membrane disruption [27]. Alkaloids are heterocyclic nitrogen complexes that are capable of inhibiting nucleic acid synthesis, via the inhibition of the enzyme dihydrofolate reductase [28]. In DFU treatments, natural extracts have been employed both in their free forms, in ointments or lotions, and loaded onto dressing systems. El-Ghoul et al. engineered a bioactive and superabsorbent cellulosic dressing grafted with alginate and *Carthamus tinctorius* extract and verified the ability of the polysaccharide extract to inhibit four different pathogenic bacteria, revealing particular effectiveness against Gram-positive bacteria [29]. Polycaprolactone electrospun meshes loaded with *Gymnema sylvestre* leaf extract also revealed great antibacterial performance via contact-mediated inhibition of Gram-positive and Gram-negative bacteria [30]. Additionally, polyurethane and carboxymethyl cellulose nanofibers containing *Malva sylvestris* extract were seen to effectively eradicate *Staphylococcus aureus* and *Escherichia coli* bacteria, without inducing any cytotoxic effect both in vitro and in vivo using diabetic mice [31].

### 3.3. Inorganic Nanoparticles

Particles of 1 to 100 nm in size are frequently denominated as nanoparticles, even though their morphology or shape can encompass spheres, capsules, liposomes, dendrimers and even micelles. They are characterized by possessing a large surface area to volume ratio and for that reason have been most sought out for biomedical purposes. Inorganic nanoparticles are subdivided into magnetic and metallic (including alloys and oxides) [32,33]. In DFU care, and particularly in fiber-based scaffolds emerging in the last five years, focus has been given to zinc oxide nanoparticles. Ahmed et al. electrospun a chitosan, poly(vinyl alcohol) and zinc oxide nanofibrous mesh and determined that antimicrobial activity was highly instigated in the presence of the inorganic particles. They also verified that by combating infection more effectively, these scaffolds were capable of accelerating healing and exhibiting a superior antioxidant profile compared to the nanoparticles-unloaded scaffolds [34]. In another study, synergisms between zinc oxide nanoparticles and the natural extract *Urtica dioica* were examined putting again in evidence the improved antibacterial potential of the engineered fibrous scaffolds. They were also found to support cell viability along with cell adhesion [35].

The inorganic nanoparticle activity against bacteria has been extensively verified, with their mechanisms of action being already detailed. In fact, inorganic nanoparticles have been shown to act against microbial cells by disrupting the cell wall and causing membrane damage, and by infiltrating through the cell membrane and inducing protein denaturation, enzyme inactivation, oxidative stress, and DNA sequence breakage [36,37]. Their small size allied to their surface charge or potential linked agents at the surface or incorporated at the core have been deemed the effectors of such successful activity.

### 3.4. Polymers: Chitosan

One of the most impactful polymers in DFUs’ infection control is chitosan. This polysaccharide made of D-glucosamine and N-acetyl-D-glucosamine units linked through β-(1-4) glycosidic linkages is obtained from chitin partial deacetylation and is characterized by its biocompatibility, biodegradability, and hemostasis capacity. Most importantly, it displays broad antimicrobial action against both bacteria and fungi. After dissolution, the chitosan amino groups present at the glucosamine units become protonated (particularly at acidic pH), which has been highlighted as the most significant effector of its antimicrobial potency. Indeed, it is via the electrostatic interactions established with the negatively charged microbial cells, which damage, increase the membrane permeability and lead to the leakage of intracellular components, that chitosan acts to inhibit the activity of the pathogens infecting DFUs [38,39]. Jeckson et al. demonstrated the ability of chitosan to protect the wound site from the effects of the most prevalent bacteria in DFUs, the Gram-positive *Staphylococcus aureus* and Gram-negative *Pseudomonas aeruginosa*, via a double-layer scaffolding system [40]. In a triple-layer nanofibrous scaffold, it was seen that without chitosan the antibacterial activity of the structure was inexistent. In fact, it was proven that the sub-layer with the highest chitosan content displayed the best bacteria inhibitory efficiency [41]. Similarly, Yang et al. immobilized hydroxypropyltrimethyl ammonium chloride chitosan, a chitosan derivative, onto the surface of poly(lactic-co-glycolic acid) nanofiber meshes and corroborated this polymer’s antibacterial abilities against the same bacteria. These scaffolds were also seen to stimulate the adhesion, spreading and proliferation of fibroblasts and keratinocytes and to generally improve wound healing [42]. Indeed, derivatives of chitosan with known antimicrobial features include hydroxypropyl chitosan, thioglycolic chitosan, carboxymethyl chitosan, *N,N,N*-trimethyl chitosan, N-(2-hydroxyl)propyl-3-trimethyl ammonium chitosan, and a variety of chitosan conjugates [38]. Even though chitosan has intervention in many immunomodulatory functions in DFUs, most applications explore its antimicrobial profile.

## 4. Immunomodulatory Agents

In DFUs, healing progression can be delayed by means of a variety of factors, most of which impact significantly the skin tissues’ ability to effectively induce an appropriate immune response [5,16]. As explained earlier (Section 2), there are many impairments to the successful healing of DFUs, some of which can be stimulated using bioactive, immunomodulatory agents within dressing systems, including fiber-based scaffolds.

### 4.1. Growth Factors

Growth factors are polypeptides capable of stimulating a variety of target cells to grow, differentiate and even alter their metabolism. They can act by paracrine (targeting nearby cells) and autocrine (targeting itself) mechanisms, inducing a complex cascade of signaling pathways. All growth factors can influence various aspects of cell performance at once [43]. In DFUs, they have been shown to affect the migration of fibroblasts and keratinocytes and collagen expression and deposition. They have also been associated with the inhibition of pro-inflammatory cytokines and the stimulation of angiogenesis and granulation tissue formation. Growth factors also prevent apoptosis pathways frequently associated with the abnormalities that occur along the stages of wound healing in DFUs [44,45]. In nanofibrous constructs, the recombinant human epidermal growth factor was found to improve fibroblast proliferation in vitro, aside from significantly instigating wound closure and re-epithelization in an in vivo full-thickness wound model [46]. Additionally, epidermal growth factor-loaded patches were deemed effective in promoting cell migration, angiogenesis, and rapid diabetic wound healing [47].

### 4.2. Blood Components: Platelet-Rich Plasma

Activated platelets are known to release growth factors and other cues responsible for inducing proliferation, angiogenesis, tissue remodeling and even modulating the inflammatory response in chronic wounds. Platelet-rich plasma, also known as autologous conditioned plasma, is a concentrated substance made of the patient’s own platelets. Because of its high content of growth factors, sometimes five to 10 times greater than the usual blood composition, it can reactivate latent endogenous regeneration mechanisms [48]. Platelet-rich plasma has been most sought after in DFUs because of its clot-inducing abilities and anti-inflammatory profile. Most importantly, platelet-rich plasma has been shown to reduce the cytotoxicity of implantable scaffolds. Indeed, Meamar et al. demonstrated that gelatin nanofibrous scaffolds modified with mesenchymal stem cells and platelet-rich plasma significantly promote wound healing. Even though the enriched plasma had little effect in instigating the different biological pathways associated with each wound healing stage, it was seen to effectively improve the biocompatibility of the scaffold [49]. It should be, however, be pointed out that platelet-rich plasma still requires further research as it has been associated with negative impacts in wound healing, which have been explained by the lack of standardizing methods for preparation and the variability between donors (conditioned by the patient’s medical history) [50].

### 4.3. Natural Extracts

From an immunomodulatory perspective, plant extracts have been widely used in traditional medicinal formulations, as they possess many bioactive compounds and metabolites: alkaloids, carotenoids, glycosides, terpenes, and flavonoids. These elements have been linked to important phenomena associated with DFUs healing, including a decrease in glucose absorption, an increase in insulin production, stimulation of fibroblasts and keratinocytes migration, proliferation, and differentiation, enhanced collagen deposition and expression, and stimulation of anti-inflammatory and antioxidant events [19,51]. Moreover, bixin, an apocarotenoid found in the seeds of the achiote tree (*Bixa orellana*), loaded onto polycaprolactone nanofibers was also found to accelerate wound healing and reduce the scar tissue area [52]. In another study, curcumin incorporated within poly(lactic acid) and hyperbranched polyglycerol nanofibers demonstrated great ability in promoting cell viability, adhesion and proliferation, while also instigating cell migration [53]. *Melilotus officinalis* extract has also been explored in a triple-layer nanofibrous scaffold made of polycaprolactone and collagen and was shown to successfully regenerate tissues of the skin in diabetic ulcers by stimulating the angiogenesis, collagen production and deposition, and re-epithelialization [54].

### 4.4. Proteins: Collagen, Silk Fibroin and Sericin, and Keratin

Collagen is a natural fibrous protein commonly found in the connective tissues and in the skin extracellular matrix (ECM). It is a biocompatible, structural, biomimetic and low immunogenic protein that has been used as a biological component in many regenerative medicine scaffolding systems by instigating the formation of cell-level interactions with living tissues (fibrillogenesis) [55]. In DFUs, collagen has been shown to stimulate the attachment, proliferation, and migration of fibroblasts and to modulate granulation tissue formation [39,55]. However, because of its low mechanical and chemical stability, it is frequently combined with other polymers for producing wound dressings. Gao et al. proposed the combination of collagen with polycaprolactone and bioactive glass nanoparticles and determined that the scaffold could improve endothelial cell attachment and proliferation, and significantly enhance angiogenesis, granulation tissue formation, collagen matrix remodeling and epidermis differentiation [56]. Another collagen-containing polycaprolactone scaffold was found to improve water uptake and cell biocompatibility, reducing dressing change frequency [57]. In combination with silk fibroin and a poly(vinyl alcohol-based scaffold), collagen was also found to promote cell attachment, spreading and proliferation [58]. Silk fibroin is a fibrous protein secreted by *Bombyx mori* silk glands, which exhibits excellent mechanical performance, controllable biodegradability, improved hemostatic profile, great biocompatibility, and low antigenicity [59,60]. Most importantly, in DFUs, it can reduce inflammation and augment the expression of transforming growth factor-β signaling pathway and collagen during wound healing, as reported by Xu et al. [61]. Additionally, modified with insulin, silk fibroin dressings can also accelerate wound closure, collagen deposition and vascularization [62]. Sericin is another protein that can be extracted from *Bombyx mori* silk glands, however, at a lower amount than silk fibroin (80% versus 20% for sericin) [59]. Silk sericin is a globular protein with remarkable biodegradability, biocompatibility, antioxidant capacity, and regenerative abilities, including faster wound healing abilities, accelerated cell proliferation, and a low inflammatory profile. Silk sericin is less explored than silk fibroin; however, fiber-based scaffolding systems for DFU care have already been engineered [59,63]. Indeed, Gilotra et al. demonstrated the antioxidant potential of silk sericin without compromising cell viability and verified its ability to accelerate wound healing without triggering any inflammatory response [64].

Keratin is a fibrous protein with excellent mechanical features. It is abundant in the skin, hair, nails, claws, horns, feathers, and hooves of many animals. In humans, keratin, from the epidermal proteins family, works as an important skin barrier element due to its structural properties. Moreover, keratin contains cell adhesion sequences, such as the arginine-glycine-aspartic acid (RGD) or the leucine-aspartic acid-valine (LDV) motifs, which can support cell attachment, spreading and proliferation [65,66]. This is exactly what was determined via Yao et al.’s experiments. They concluded that by generating a double-layer keratin-loaded gelatin mesh they could accelerate cell attachment and proliferation above commercial dressing systems [67]. In addition, keratin has been shown to improve the biocompatibility of wound-healing scaffolds, while guaranteeing their structural stability for sustained drug delivery [68].

### 4.5. Neuropeptides

Neuropeptides consist of short-sequence amino acids that work to modulate synaptic activity or as primary neurotransmitters, activating the immune system and cell proliferation during the healing process. Neuropeptides such as calcitonin gene-related peptide, neuropeptide Y, neurotensin, and α-melanocorticotropin-releasing hormone have been identified as biomarkers in DFUs by promoting the expression of interferon-β, transforming growth factor-β, and the macrophage inflammatory protein-1α [45,69]. Zheng et al. even showed that polylactide-polyglycolide and cellulose nanocrystals fibrous meshes loaded with neurotensin are capable of inducing epidermal and dermal regeneration and increasing the ratios of the fibrotic area to the whole affected region. These neuropeptide-containing scaffolds were also seen to reduce the expression of the inflammatory cytokines interleukin-1β and interleukin-6 [70].

### 4.6. Stem Cells

Stem cells are the body’s raw matter, from which other cells and tissues with specialized functions can be generated. They can be subdivided into embryonic, derived from the inner cell mass of a blastocyst, and adult stem cells, which can be obtained from bone marrow, peripheral blood, and hair follicles [39,71]. In DFUs, stem cells can repopulate lost or injured areas with differentiated cells (i.e., keratinocytes, endothelial cells), aiding the self-renewal of tissues due to their multipotency, or promoting the recruitment of inflammatory cells through paracrine secretions (i.e., cytokines, chemokines, growth factors, and extracellular vesicles containing proteins, mRNA, microRNAs, and mitochondria) [72,73]. Indeed, stem cells have been shown to migrate into injured sites and actively work towards the elimination of infections and to promote tissue homeostasis, aside from enhancing angiogenesis, accelerating re-epithelialization, and instigating granulation tissue formation [73]. Stem cells have been mostly employed in full-thickness wound treatments, both as free, topically delivered agents and as part of scaffolding systems, including fiber-based constructs. Chen et al. demonstrated that by engineering a 3D scaffold consisting of radially or vertically aligned nanofibers modified with bone marrow mesenchymal stem cells. They generated a personalized platform, adaptable to all DFUs’ patient needs, that significantly enhanced angiogenesis and ECM deposition, promoted granulation tissue formation and, generally, elicited a pro-regenerative response from the wounded tissues [74]. Nanofibrous meshes containing human placenta-derived mesenchymal stem cells and platelet-rich plasma were also found to induce cell recruitment and proliferation to the injured area, and to stimulate wound closure, while reducing pain [49]. More recently, micropatterned fiber scaffolds prepared via the electrohydrodynamic cryo-printing method and bonded with adipose-derived mesenchymal stem cells were seen to improve the secretion of growth factors and chemokines, thus instigating fibroblast migration and vascular endothelial cell tube formation, along with collagen deposition and angiogenesis and reducing pro-inflammatory reactions [75].

### 4.7. Polymers: Alginate and Hyaluronic Acid

Alginate is an anionic polysaccharide made of b-L-guluronic acid and (1–4) related to a-D-mannuronic acid and extracted from brown seaweed. It is a biodegradable, biocompatible, non-toxic polymer known to promote oxygen permeability and hemostasis and to reduce odor and pain in wounds. Its most attractive features for DFU healing rely on its hemostat, gel-forming, highly exudate-absorbent abilities, which guarantee local moisture balance, stimulating re-epithelialization and granulation [51,76]. Additionally, alginate has been shown to decrease pro-inflammatory cytokines expression and inhibit free radicals’ formation via elastase binding, an enzyme that becomes highly expressed in chronic wound scenarios [8,77]. In DFUs, a three-layered nanofiber wound dressing has been shown to inhibit matrix metalloproteinase-2 effect via doxycycline, improve remodeling via collagen, and guarantee proper wettability, absorption capacity and bio-adhesion due to the combinatory effects of chitosan and alginate [78]. A similar effect was demonstrated by Anand et al.’s scaffolds made of polyvinyl alcohol (PVA), sodium alginate and silk fibroin, in which alginate was found to guarantee the wound site moisture control, with critical water retention for effective drug delivery and wound healing [79].

Hyaluronic acid is a hygroscopic glycosaminoglycan made of repeating polymeric disaccharides of D-glucuronic acid and N-acetyl-D-glucosamine linked through a glucuronidic β (1–3) bond. It is characterized by its biocompatibility, biodegradability, hydration and lubrication capacities, anti-adhesive and bioresorption features, and its viscoelasticity [51]. It is mostly found in the connective tissues of the ECM. Considering its abundance in the skin, accounting for 50% of the total body presence of hyaluronic acid, it is frequently employed in dressing systems to treat DFUs. Aside from its high-water uptake capacity which prevents wound dryness, hyaluronic acid is known to increase collagen secretion via fibroblast migration and proliferation and to activate inflammatory cells towards an enhanced immune response. It has also been shown to contribute to angiogenesis [80,81]. In a core-shell nanofiber scaffold made of polyethylene oxide, polycaprolactone, keratin and hyaluronic acid, this biopolymer was seen to guarantee great swelling capacity, fast degradation and increased cumulative drug release, aside from enhancing the scaffold biocompatibility in vitro [68]. Similar observations were made with core-shell nanofibrous structures made of polyurethane, starch and hyaluronic acid. This biopolymer wound healing impact was also confirmed in vivo, by contributing to accelerated cell repair [81].

## 5. Advanced Fibrous Scaffolds

**Figure 3:**
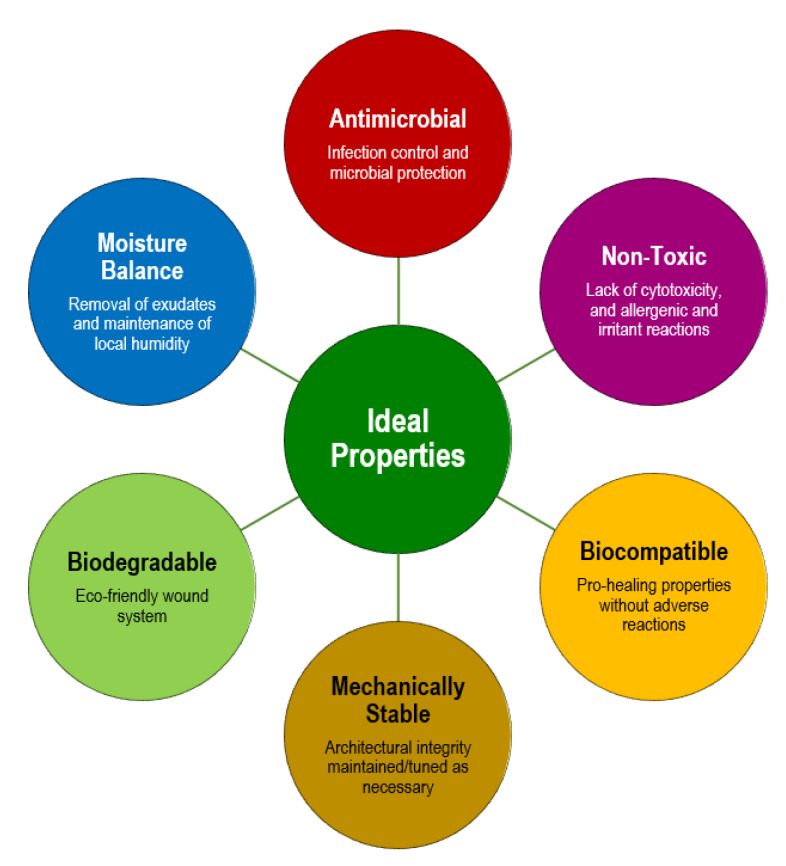
Requirements for the formulation of a scaffolding system for applications in DFUs therapies.

Limitations in the ECM (cell defects, protein degradation, dysregulated enzyme activity, etc.), accumulation of devitalized/necrotic tissues and appearance of infections caused by polymicrobial wound bed invasion and colonization are the main characteristics of non-healing DFUs [8,82]. Advanced fibrous scaffolding systems may aid in restoring functions and replacing missing ECM and fighting pathogens. Ideally, these constructs should entail properties that closely mimic the naturally occurring elements that they are trying to resemble or pass for, provide physical and mechanical stability for quick integration, restoration of functions and protection from additional harm, and release chemical cues or bioactive agents that will actively work to protect the region against invaders and/or trigger events that will promote cell recruitment and the progression of the healing cascade (Figure 3) [83].

Three-dimensional fibrous scaffolds, with a porous structure and small fiber diameters (mostly in the nanoscale) are gaining immense popularity and applicability in DFU treatments. Their similarity to the ECM structure, particularly those scaffolding systems produced with a random fiber arrangement, can closely mimic the fibrous entangled organization of the ECM proteins, offering many advantages for cell recruitment, attachment, and proliferation. Among the many techniques used to produce fiber-based scaffolds for DFU therapies, the electrospinning approach is the most employed. This technique allows the control of several parameters during mesh production, namely porosity, pore size, fiber diameter, alignment, etc., and is cost-effective, versatile, and easy to process. Most importantly, it allows for the production of fibers on the nanometer scale, whose arrangement guarantees effective gas permeation (oxygen transfer), and displays a large surface area for effective binding with bioactive agents or cells (depending on the intended functions) or for the sustained liberation of agents pre-loaded into the polymeric solution prior to nanofiber mesh production [8,84]. As seen in Table 1, electrospun nanofiber scaffolds of single and multiple layers have an impactful place in therapeutic formulations for DFU care. They can be modified with a variety of bioactive agents for inducing antimicrobial and/or immunomodulatory effects. Many reports have exposed formulations with natural-origin polymers (i.e., chitosan, gelatin, sodium alginate, cellulose, etc.), synthetic polymers (i.e., polycaprolactone, poly(vinyl alcohol), polylactic-co-glycolic acid, poly(lactic acid), etc.), or combinations of both. Extensive revision work has been conducted on the properties, characteristics, and benefits of using each of these polymers [85,86,87,88]. Additionally, other techniques such as aqueous phase fiber reassembly [57] and cryoprinting [75] may also be used in the production of 3D fibrous scaffolding systems with great biological performance. Textile knitting is another very common technique for bandage production applied to DFUs care [29,89], either as a main (Table 1 only reports studies in which knitted fiber constructs are the main therapy) or a co-adjuvant, protective therapy.

## 6. Conclusions

DFUs are very complex chronic wounds, demanding active strategies to manage and treat the affected regions. Polymers processed in the form of fibers and modified with bioactive agents have been proven to have great efficiency in generating environments conducive to wound healing, with exceptional outcomes both in vitro and in vivo. Among the many technical approaches available for fiber-based scaffolding systems production for DFUs care, electrospinning has taken the lead, gaining much interest for its ability in generating scaffolds with improved porosity and air and water-vapor permeabilities essential for cell growth, attachment and proliferation; high water intake and hydration capacity which guarantees local moisture balance; good mechanical stability and tunable flexibility for appropriate adaptation to the injured site, offering protection, and tunable fiber size diameters (micro to nanoscale), alignment (random, aligned) and chemical composition so resemblances can be drawn to the skin ECM and, hence, facilitate recognition and integration of the scaffolding system. Most importantly, these structures allow the integration of bioactive compounds or chemical cues that actively intervene in different stages of the healing of these diabetic wounds, accelerating injury recovery by promoting vascularization, collagen deposition, and the restoration of physiological functions. Both antimicrobial and immunomodulatory agents have been shown to play decisive roles in DFU care, actively contributing to the healing cascade. In fact, many of the formulations that combine these bioactive agents and fiber constructs described in this work have already been tested against commercial products demonstrating improved efficacy. Considering the Nanospider™ technology is the only available option for electrospinning these nanofibrous systems at an industrial scale, investments should be conducted to optimize these approaches, either by approximating laboratory-scale testing to the available technology or by improving on the pre-existent technology, augmenting the features available for processing. Nowadays, much investment is also being conducted in seamless technology for bandage production, 3D printing for fiber patterned constructs, or even on wet-spun systems [103]. This continued research for new, faster healing and more target-directed dressings is essential for efficient treatment strategies, adaptable for each situation and patient, and plays major roles in the physical and psychological states of patients dealing with DFUs.

## Figures and Tables

**Table 1:** Examples of fiber-based systems containing bioactive components of different origins with potential for applications in DFU care and detailed analyses of their biological contributions (antimicrobial and immunomodulatory). Data report work published only within the last five years (2017–2022).

Technique	Final System	Components	Main Bioactive Agents Categories	DFUs Care Potential	Ref.
Electrospinning	Triple Layer Mesh	Outer and middle layers: sodium alginate (SA) and chitosan (CS); Inner layer: co-axial nanofibers with core made of polycaprolactone and collagen, and shell of doxycycline and polyethylene oxide	CS (polymer), collagen (protein) and doxycycline (antibiotic); each layer contained one main inducer of biological activity	- Porous, flexible, mechanically resilient and wettable aligned fiber meshes were produced; - Absence of cytotoxic effects against keratinocyte cells even with quick release of drug; - Guaranteed prolonged protection of the payload (12 months); - Expression of matrix metalloproteinase-2 enzyme was inhibited, and ECM remodeling was promoted.	[78]
		Outer layer: polycaprolactone (PCL) Middle layer: PCL and type I collagen Inner layer: type I collagen and *Melilotus officinalis* extract	Collagen (protein) and *Melilotus officinalis* extract (plant extract)	- Smooth and defect-free three-layer nanofiber structures were obtained; - In vitro cell studies verified the fibroblast cells viability; - 18-day in vivo studies demonstrated the scaffold to induce proper re-epithelialization in diabetic wounds and to instigate collagen production and deposition in the newly formed skin.	[54]
		Outer layer: PCL Middle layer: polyvinyl pyrrolidone (PVP) and ciprofloxacin Inner layer: poly(acrylic acid) (PAA)	Ciprofloxacin (antibiotic)	- The scaffolding system displayed great mechanical resilience, with the PCL outer layer exhibiting lower wettability, adherence, and moisture uptake than the remainder layers; - Drug release was guaranteed in a sustained manner and was influenced by loading amount on the scaffold.	[90]
		Outer layer: poly(vinyl alcohol (PVA) and nanobioglass (nBG) Middle layer: CS and PVA Inner layer: CS	nBG (inorganic particles) and CS (polymer)	- The multilayer mesh exhibited excellent biocompatibility, antibacterial activity and regenerative effects; - Each layer allowed for a microenvironment to be generated so optimal performances of each component could be attained; - In vivo wound model revealed the mesh to significantly accelerate and enhance healing, in terms of complete re-epithelialization, improved collagen alignment and formation of skin appendages.	[41]
	Double Layer Mesh	Inner layer: gelatin (GN) with keratin Outer layer: polyurethane (PU)	Keratin (protein)	- Nanofibers presented a uniform morphology and bead-free structure; - Fibroblast-like cells attachment was improved (increased cell spreading) and proliferation was accelerated; - Engineered meshes promoted faster vascularization, facilitating wound repair and growth of thicker epidermis.	[67]
		Inner layer: CS, PVA and deferoxamine Outer Layer: SA and PVA	CS (polymer) and deferoxamine (immunomodulatory drug)	- The double layer dressing displayed high swelling degree, sufficient water-vapor permeation, and high drug entrapment efficiency with a sustained release up to 48 h; - Meshes antibacterial effectiveness was confirmed; - The dressing was also deemed cytocompatible, with in vitro scratch testing revealing wound healing potential.	[40]
	Core-Shell Nanofibers	Shell: polyethylene oxide (PEO) and PCL Core: hyaluronic acid (HA), keratin and metformin hydrochloride	HA (biopolymer), keratin (protein) and metformin hydrochloride (biguanide antihyperglycemic agent)	- Shell structure guaranteed a prolonged released of the core-entrapped bioactive agents; - In vitro cell cultures demonstrated the enhanced biocompatibility of the engineered core-shell fibers and attested to their efficacy as drug delivery platforms.	[68]
		Shell: PEO, CS and vancomycin Core: PVP, GN and imipenem/cilastatin	CS (polymer) and vancomycin and imipenem/cilastatin (antibiotics)	- Core-sell nanofibers displayed a smooth morphology with no cytotoxic evidence against fibroblastic cells; - Shell structure protected antibiotic at the core, guaranteeing a prolonged liberation, while the antibiotic at the shell experienced a faster release; - Nanofibers exhibit significant antibacterial profile against bacteria prevalent in DFUs.	[23]
		Shell: PU Core: starch and HA	HA (biopolymer)	- Porous core-shell structures were obtained; - In vitro cell morphology, viability and attachment were enhanced in the presence of the HA-loaded scaffold; - In vivo studies demonstrated the scaffolds to accelerate wound healing.	[81]
		Shell: poly-D-L-lactide-glycolide (PLGA) Core: insulin	Insulin (peptide hormone)	- Core-shell fibers sustained the release of insulin for 4 weeks, guaranteeing a balanced moist environment as well compared to single layer meshes; - Nanofibers reduced the amount of type I collagen in vitro but increased the transforming growth factor-beta (TGF-β) content in vivo and promoted diabetic wound repair.	[91]
		Shell: PLGA, vancomycin and gentamycin Core: recombinant human platelet-derived growth factor-BB (rhPDGF-BB)	Vancomycin and gentamycin (antibiotics) and rhPDGF-BB (growth factor)	- Scaffolds guaranteed a sustained release of growth factor and antibiotics for 3 weeks; - They also decreased phosphatase and tensin homolog content, enhanced angiogenesis marker presence (CD31), and accelerated healing in early-stage infected diabetic wounds.	[22]
	Single Layer Mesh	PLGA modified with recombinant human epidermal growth factor (rhEGF) and *Aloe vera* extract	rhEGF (growth factor) and *Aloe vera* (plant extract)	- Uniform, bead free meshes of improved porosity were obtained; - Presence of rhEGF and the extracts improved fibroblast proliferation and accelerated significantly wound closure and reepithelization in an in vivo full thickness wound mice model.	[46]
		GN modified with human placenta-derived mesenchymal stem cells (hPDMSCs) and platelet-rich plasma (PRP)	hPDMSCs (stem cells) and PRP (blood components)	- Clinical testing was conducted on 28 patients with DFUs, from which 18 were treated with the smooth, homogenous nanofibers mats with and without PRP; - Cell proliferation and wound closure were significantly enhanced by hPDMSCs, however, PRP had little impact on the outcomes; - Pain was significantly reduced in the presence of the engineered nanofibers as compared to conventional standard care therapies (control group).	[49]
		PCL and GN composite containing silicate-based bioceramic particles of nagelschmidtite (NAGEL)	NAGEL (inorganic particles)	- The scaffolding system promoted the adhesion, proliferation and migration of human umbilical vein endothelial cells (HUVECs) and human keratinocytes (HaCaTs) in vitro; - In vivo evaluations demonstrated their ability in inducing angiogenesis, collagen deposition and re-epithelialization, as well as in inhibiting inflammation.	[92]
		Hydroxypropyl methylcellulose (HPMC) and PEO loaded with β-glucan	β-glucan (immunomodulatory drug)	- Electrospinning Nanospider™ technology ensure the reproducible and reliable production of nanofibers; - The scaffolds exhibited no cellular toxicity in vitro; - Wound healing assessment in a wound model confirmed the significant improvement introduced by βG-nanofibers.	[93]
		Poly(L-lactic acid) (PLLA) and dimethyloxalylglycine-loaded mesoporous silica nanoparticles (MSi NPs)	Dimethyloxalylglycine (immunomodulatory drug) and MSi NPs (inorganic particles)	- The engineered aligned porous meshes stimulated the proliferation, migration and angiogenesis-related gene expression of endothelial cells; - In vivo study demonstrated the meshes’ ability to improve neo-vascularization, re-epithelialization and collagen formation, while inhibiting inflammatory reactions in the diabetic wound bed.	[94]
		GN, arabinoxylan ferulate (AXF) and silver sulfadiazine	AXF (polysaccharide) and silver sulfadiazine (inorganic compound)	- Continuous, homogeneous fibers were attained with excellent biocompatibility and antimicrobial profiles; - Prolonged liberation of silver compound was guaranteed potentiating the scaffold testing in in vivo scenarios.	[95]
		ECM-componential collagen, PCL and bioactive glass nanoparticles (BGNs)	Collagen (protein) and BGNs (inorganic particles)	- Endothelial cell attachment and proliferation were enhanced; - Angiogenesis marker CD31 expression was upregulated in vitro; - Angiogenesis was also improved in vivo, by greatly upregulating the mRNA and protein expressions of hypoxia-inducible factor 1-α (Hif-1α), vascular endothelial growth factor (VEGF), collagen I and α-smooth muscle actin (α-SMA); - Granulation tissue formation, collagen matrix remodeling and epidermis differentiation were accelerated.	[56]
		Bixin-loaded PCL nanofibers	Bixin (carotenoid pigment extracted from *Bixa orellana* L. seeds)	- Increasing bixin concentration resulted in higher polymeric solution electrical conductivity and, consequently, in smaller fiber diameters; - Bixin release kinetics was guaranteed for 14 days (30–40% release in the first 10 h); - The bixin-loaded meshes accelerated wound healing while reducing the scar tissue area.	[52]
		PCL and *Gymnema sylvestre*	*Gymnema sylvestre* (plant extract)	- Scaffolds exhibited good wettability and enhanced mechanical properties; - Contact-mediated bacterial inhibition was achieved against Gram-positive and Gram-negative bacteria; - Scaffolds were identified as cytocompatible towards fibroblasts.	[30]
		PU and carboxymethyl cellulose (CMC) nanofibers containing *Malva sylvestris* extract	*Malva sylvestris* (plant extract)	- Meshes allowed great fluid absorption and sustained release of the extract; - Significant antibacterial activity was observed; - In vivo wound-healing testing indicated the scaffold accelerated healing significantly, aside from lowering acute and chronic inflammations; - Collagen deposition and neovascularization were also instigated.	[31]
		Poly(lactic acid) (PLA) and hyperbranched polyglycerol (HPG) modified with curcumin	Curcumin (plant extract)	- Meshes were deemed highly hydrophilic, absorbent and with great drug uptake; - In vitro cell viability, adhesion and proliferation were instigated as well as cell migration (scratch test).	[53]
		Neurotensin-loaded PLGA and cellulose nanocrystals (CNCs) composite	Neurotensin (neuropeptide)	- PLGA/CNCs nanofibers showed excellent cytocompatibility and facilitated fibroblast adhesion, spreading and proliferation; - Neurotensin could be released from the mats in a sustained manner for up 2 weeks; - In vivo data reported the composite abilities to induce faster epidermal and dermal regeneration, while decreasing the expressions of the inflammatory cytokines interleukin-1β (IL-1β) and IL-6.	[70]
		GN and bacterial cellulose (BC) modified with metformin and glybenclamide	Metformin and glybenclamide (diabetic drugs)	- Nanofibers were produced using a portable electrohydrodynamic gun with great homogeneity; - Diabetic wounds treated with nanofibers loaded with glybenclamide revealed moderate to complete re-epithelialization and well-formed granulation tissue, a result superior to the metformin-modified fibers; - TNF-α levels were significantly reduced with both drugs, but again glybenclamide was more effective.	[96]
		PLA and doxycycline	Doxycycline (antibiotic)	- Antibiotic homogeneous distribution along the fibers for a sustained release was achieved; - The mats’ mechanical features, water-vapor permeability and absorbency met the requirement for wound dressings; - In vitro data confirmed the mats’ cytocompatibility and antibacterial profile; - In vivo full-thickness wound healing was also stimulated.	[21]
		PVA incorporating active silk sericin	Silk sericin (protein)	- Dressings were endowed with free radical scavenging capacity, antibacterial activity, swelling capacity, and biocompatibility due to the incorporation of silk sericin; - Fibroblasts and keratinocytes spreading and proliferation were improved; - The nanofibers exhibited excellent antioxidant potential without hampering cell viability even under H_2_O_2_ driven oxidative stress; - In vivo tolerance to the engineered dressing was confirmed over 4 weeks of testing, with no inflammatory events being triggered.	[64]
		CS, PVA and zinc oxide nanoparticles (ZnO NPs)	ZnO NPs (inorganic particles)	- CS/PVA/ZnO nanofibrous meshes possessed exceptional antibacterial activity against DFUs-prevalent bacteria; - They also exhibited excellent antioxidant potential; - In vivo wound healing studies showed that CS/PVA/ZnO meshes accelerated wound healing.	[34]
		PCL, ZnO NPs and *Urtica dioica*	ZnO NPs (inorganic particles) and *Urtica dioica* (plant extract)	- Incorporation of *Urtica dioica* and ZnO NPs improved the fibers’ water uptake and controlled the release of the plant extract; - Antibacterial activity was augmented and cell cytotoxicity was diminished in the presence of the hybrid scaffold; - Cell adhesion and, consequent, scaffold integration was instigated.	[35]
		PVA, astragalus and astragaloside IV liposomes.	Astragalus (polysaccharide, plant extract) and astragaloside IV (cycloartane-type triterpene obtained from *Astragalus membranaceus*)	- In vivo testing revealed the nanofibers to inhibit inflammation, enhance deposition of collagen and the repair of regenerated epithelium, and effectively strengthen wound healing of diabetic rats.	[97]
		PVA, SA and silk fibroin (SF) fibers loaded with asiaticoside.	SF (protein) and asiaticoside (pentacyclic triterpenoid isolated from *Centella asiatica* plant)	- Homogeneous nanofibers with sustained asiaticoside liberation over extended periods were electrospun; - Mats exhibited low cytotoxicity, promoting improved cell migration and anti-microbial efficacy; - In vivo testing revealed wound healing efficacy and abilities to restore normal skin structure.	[79]
		PVA, SF, type I collagen and S-Nitrosoglutathione	SF (protein), type I collagen (protein) and S-Nitrosoglutathione (nitric oxide donor)	- Continuous, bead free and randomly oriented nanofibers meshes were obtained with a highly porous morphology; - In vitro evaluations attested to the scaffold biocompatibility with a high level of cell attachment, expansion, inter-cellular connections and proliferation, mainly promoted by type I collagen; - Nitric oxide release, essential for effective wound healing, was guaranteed for 1 day.	[58]
		Poly-(L-lactide-co-caprolactone) (PLCL) and SF loaded with Huangbai Liniment	SF (protein) and Huangbai Liniment (plant extract)	- Smooth and bead-free nanofibers allowed for a sustained release of the natural-origin drug; - Antibacterial effects were observed against Gram-positive and Gram-negative bacteria; - In vitro cell adhesion and proliferation were enhanced; - In vivo testing demonstrated the loaded nanofibers to instigate the expression of the TGF-β signaling pathway and collagen and to inhibit pro-inflammatory factors, thus effectively promoting healing.	[61]
Electrospinning and Entrapment-Graft	Single Layer Mesh	PLGA-hydroxypropyltrimethyl ammonium chloride chitosan (HACC) composite	HACC (polymer)	- Effective antibacterial activity towards both Gram-positive and Gram-negative bacteria; - Meshes were cytocompatible, significantly stimulating adhesion, spreading and proliferation of fibroblasts and keratinocytes; - PLGA-HACC exhibited excellent wound healing efficacy in vivo.	[42]
Electrospinning combined with a Spray Phase-Inversion Method	Double-Layer Meshes	PU combined with fibrin fibers loaded with platelet lysate	Fibrin (protein) and platelet lysate (blood component)	- The bilayer dressing allowed a sustained release of bioactive platelet-derived growth factors; - The engineered scaffold also significantly accelerated wound closure in in vivo full-thickness wounds; - Histological data demonstrated the scaffold’s effectiveness in increasing re-epithelialization and collagen deposition.	[98]
Electrospinning followed by Hydrogel Loading	Fiber-Hydrogel Composite	PLA nanofibers loaded with HA, valsartan, and ascorbic acid hydrogel	HA (biopolymer), valsartan (immunomodulatory drug) and ascorbic acid (vitamin C)	- Scaffolds offered a large surface area for enhanced drug solubility, oxygen permeability, and fluid uptake; - Presence of valsartan significantly impacted the re-epithelization rate, accelerating it; - Scaffolds also reduced the number of inflammatory cell infiltrates at the wound site.	[99]
		Poly(3-hydroxybutyrate-co-3-hydroxyvalerate) (PHBV) nanofibers modified with GN-methacryloyl (GNMA) hydrogels containing epidermal growth factors (EGFs)	EGFs (growth factors)	- Patches promoted cells migration and proliferation and enhanced angiogenesis in vitro; - In vivo wound healing testing in diabetic rats showed the patches stimulated favorable cell responses, angiogenesis and rapid wound healing.	[47]
		SA, SF and amniotic fluid	SF (protein) and amniotic fluid (complex substance containing a wide range of growth factors)	- SF fibers were successfully linked to the SA-based hydrogel formulation; - Sustained release of amniotic fluid was guaranteed at the optimal ratio with SA (superior amount of amnionic fluid); - Fibroblast-like cells proliferation, spreading, and secretion of collagen were enhanced with increasing concentrations of amniotic fluid.	[100]
Electrospinning followed by Cryogenic Cutting and Thermal Treatment	Cylindrical 3D Scaffolds made of Radially or Vertically Aligned Nanofibers	PCL, GN and Pluronic-F-127, modified with bone marrow mesenchymal stem cells (BMSCs)	BMSCs (stem cells)	- Scaffolds can be customized with different sizes, depths, and shapes for a variety of type 2 diabetic wounds; - They were also shape-recoverable in the atmosphere and water following compression; - Enhanced the formation of granulation tissue, promoting angiogenesis, and facilitating collagen deposition; - Inhibited the expression of pro-inflammatory cytokines IL-6 and tumor necrosis factor-α (TNF-α) and promote the expression of anti-inflammatory cytokines IL-4 and IL-10.	[74]
Electrospraying	Fibrous Sponge Functionalized with Co-axial Microparticles	Insulin-encapsulated SF microparticles loaded onto SF sponge	SF (protein)	- SF microparticles guaranteed the sustained release of insulin for up to 28 days; - Insulin retained its bioactivity and promoted cell migration; - The engineered dressing was seen to accelerate wound closure, collagen deposition and vascularization.	[62]
Weft Knitting	Three-Layer Fabric	Polyethylene terephthalate (PET) and PU yarns modified with quaternary ammonium salt (QAS)	QAS (cationic salt; organic particles)	- Effective exudates management, with optimal moisture balance, and oxygenation of the wound (porous nature); - Excellent broad-spectrum antimicrobial activity, with non-leaching of salts, thus preventing the development of mutations or microbial resistance; - Durable and cost-effective dressing.	[89]
Knitting followed by Grafting (adjustable curing and impregnation conditions)	Cellulosic Textile Woven	Cellulosic textile woven grafted with alginate and *Carthamus tinctorius* polymer extract	Alginate (polymer) and *Carthamus tinctorius* polymer extract (plant extract)	- Grafting was successfully conducted without compromising textile properties; - Excellent biocompatibility, with increased cell viability after grafting; - Antimicrobial testing against four of the most prevalent DFU bacteria demonstrated the engineered dressing potent antimicrobial effect.	[29]
Tappi T 205 sp-02 (standard compression method of pulp and paper industry)	Fibrous paper	Cellulose fibers from bleached softwood Kraft lignin modified with silver phosphate	Silver phosphate (inorganic salt)	- Efficient antimicrobial activity against *Staphylococcus aureus* bacterium; - Antimicrobial effectiveness equal or superior to commercial products.	[101]
Aqueous Phase Fiber Reassembly	3D Fibrous Scaffold	PCL and collagen nanofibers loaded with doxycycline hyclate-modified halloysite nanotubes and cephalexin	Collagen (protein), doxycycline hyclate (tetracycline antibiotic), halloysite nanotubes (inorganic nanostructures made of alumina-silicate) and cephalexin (antibiotic)	- The scaffold exhibited high water absorption capacity and swelling capacity, potentially reducing dressing change frequency in DFUs; - It also displayed excellent antibacterial activity against *Escherichia coli* and *Staphylococcus aureus* bacteria; - Additionally, the dressing demonstrated good biocompatibility, significantly improving wound healing.	[57]
Electrohydrodynamic Cryoprinting	Micropatterned fiber scaffolds	PC loaded with adipose-derived MSCs (AMSCs)	AMSCs (stem cells)	- Efficient pore formation along the scaffold for increased surface roughness (facilitated cell adhesion); - In vitro enhanced secreting of growth factors and chemokines, which promoted fibroblast migration and vascular endothelial cell tube formation; - Augmented scarless collagen deposition and angiogenesis, and reduction of pro-inflammatory reactions in in vivo experimentation.	[75]
Pressurized Gyration	Single Layer Mesh	PVP and PCL loaded with pioglitazone hydrochloride (PHR)	PHR (immunomodulatory drug)	- PHR-loaded fibrous mats expedited diabetic wound healing in type-1 diabetic rats without triggering any cytotoxic effect on cells; - Additionally, mats improved neutrophil infiltration, edema, and reduced inflammation, aside from increasing epidermal regeneration and fibroblast proliferation.	[102]

## Data Availability

Not applicable.
